# Development and Validation of an XGBoost-Algorithm-Powered Survival Model for Predicting In-Hospital Mortality Based on 545,388 Isolated Severe Traumatic Brain Injury Patients from the TQIP Database

**DOI:** 10.3390/jpm13091401

**Published:** 2023-09-19

**Authors:** Yang Cao, Maximilian Peter Forssten, Babak Sarani, Scott Montgomery, Shahin Mohseni

**Affiliations:** 1 Clinical Epidemiology and Biostatistics, School of Medical Sciences, Faculty of Medicine and Health, Örebro University, 701 82 Orebro, Sweden; scott.montgomery@oru.se; 2 Unit of Integrative Epidemiology, Institute of Environmental Medicine, Karolinska Institutet, 171 77 Stockholm, Sweden; 3 Department of Orthopedic Surgery, Örebro University Hospital, 701 85 Orebro, Sweden; maximilian.forssten@oru.se; 4 School of Medical Sciences, Örebro University, 701 82 Orebro, Sweden; mohsenishahin@yahoo.com; 5 Center of Trauma and Critical Care, George Washington University, Washington, DC 20037, USA; bsarani@mfa.gwu.edu; 6 Clinical Epidemiology Division, Department of Medicine, Solna, Karolinska Institutet, 171 77 Stockholm, Sweden; 7 Department of Epidemiology and Public Health, University College London, London WC1E 7HB, UK; 8 Division of Trauma, Critical Care & Acute Care Surgery, Department of Surgery, Sheikh Shakhbout Medical City, Mayo Clinic, Abu Dhabi P.O. Box 11001, United Arab Emirates

**Keywords:** traumatic brain injury, Trauma Quality Improvement Program (TQIP), machine learning, prediction model, survival analysis, extreme gradient boosting (XGBoost)

## Abstract

Background: Traumatic brain injury (TBI) represents a significant global health issue; the traditional tools such as the Glasgow Coma Scale (GCS) and Abbreviated Injury Scale (AIS) which have been used for injury severity grading, struggle to capture outcomes after TBI. Aim and methods: This paper aims to implement extreme gradient boosting (XGBoost), a powerful machine learning algorithm that combines the predictions of multiple weak models to create a strong predictive model with high accuracy and efficiency, in order to develop and validate a predictive model for in-hospital mortality in patients with isolated severe traumatic brain injury and to identify the most influential predictors. In total, 545,388 patients from the 2013–2021 American College of Surgeons Trauma Quality Improvement Program (TQIP) database were included in the current study, with 80% of the patients used for model training and 20% of the patients for the final model test. The primary outcome of the study was in-hospital mortality. Predictors were patients’ demographics, admission status, as well as comorbidities, and clinical characteristics. Penalized Cox regression models were used to investigate the associations between the survival outcomes and the predictors and select the best predictors. An extreme gradient boosting (XGBoost)-powered Cox regression model was then used to predict the survival outcome. The performance of the models was evaluated using the Harrell’s concordance index (C-index). The time-dependent area under the receiver operating characteristic curve (AUC) was used to evaluate the dynamic cumulative performance of the models. The importance of the predictors in the final prediction model was evaluated using the Shapley additive explanations (SHAP) value. Results: On average, the final XGBoost-powered Cox regression model performed at an acceptable level for patients with a length of stay up to 250 days (mean time-dependent AUC = 0.713) in the test dataset. However, for patients with a length of stay between 20 and 213 days, the performance of the model was relatively poor (time-dependent AUC < 0.7). When limited to patients with a length of stay ≤20 days, which accounts for 95.4% of all the patients, the model achieved an excellent performance (mean time-dependent AUC = 0.813). When further limited to patients with a length of stay ≤5 days, which accounts for two-thirds of all the patients, the model achieved an outstanding performance (mean time-dependent AUC = 0.917). Conclusion: The XGBoost-powered Cox regression model can achieve an outstanding predictive ability for in-hospital mortality during the first 5 days, primarily based on the severity of the injury, the GCS on admission, and the patient’s age. These variables continue to demonstrate an excellent predictive ability up to 20 days after admission, a period of care that accounts for over 95% of severe TBI patients. Past 20 days of care, other factors appear to be the primary drivers of in-hospital mortality, indicating a potential window of opportunity for improving outcomes.

## 1. Introduction

Traumatic brain injury (TBI) represents a significant global health challenge, resulting in substantial mortality, morbidity, and long-term disability [1,2,3,4,5,6]. It is estimated that around 69 million individuals experience a TBI each year, worldwide. Among these cases, approximately 60,000 individuals in the United States and 82,000 individuals in Europe succumb to TBI-related fatalities annually [4,5,6]. The impact of TBI extends beyond individual patients, affecting families, communities, and healthcare systems [7,8,9,10]. Therefore, accurate prediction of adverse outcomes in TBI, particularly mortality, is of paramount importance to optimally guide patient care and rehabilitation [11,12]. Conventionally, prognostication in TBI has relied on established clinical scoring systems such as the Glasgow Coma Scale (GCS) and the Abbreviated Injury Scale (AIS) [13,14,15,16]. While these tools provide valuable information, on their own they struggle to capture the complexity of the multifaceted nature of TBI, which limits their prognostic ability in terms of accuracy and individualized risk assessment [15]. This has prompted researchers and clinicians to explore alternative approaches that leverage the power of machine learning algorithms to improve predictive models in the field of TBI [17,18,19,20,21,22].

In recent years, machine learning techniques have emerged as powerful tools for predictive modeling, including in medical domains [23,24]. Among these techniques, extreme gradient boosting (XGBoost) has gained considerable attention due to its ability to efficiently manage complex relationships, nonlinear interactions, and high-dimensional data [25,26]. XGBoost is an ensemble learning algorithm that combines predictions made by weak learners, such as decision trees, to generate a robust and accurate final prediction. By effectively integrating multiple models, XGBoost can capture subtle patterns and interactions in the dataset, leading to improved performance and generalizability [25,26]. Against this backdrop, this paper aims to implement the XGBoost technique in regression models for survival outcomes to develop and validate a predictive model for in-hospital mortality in patients with isolated severe traumatic brain injury. By leveraging the capabilities of XGBoost and incorporating a comprehensive set of demographic, admission, and clinical characteristics, the goal is also to identify the most influential predictors contributing to mortality due to traumatic brain injury.

## 2. Materials and Methods

### 2.1. Source of the Data

In total, 545,388 patients between 2013 and 2021 were included in the current study from the American College of Surgeons Trauma Quality Improvement Program (TQIP) database. The dataset was split into a training dataset, which included 80% of the patients who were used for model development and training, and an external test dataset, which included 20% of the patients to test the final model. There were no significant differences observed in the features when comparing the training and test datasets (see Supplemental Table S1).

The requirement for ethical approval was waived for the current study as it was only performed using an anonymized, retrospective dataset. The Transparent Reporting of a Multivariable Prediction Model for Individual Prognosis or Diagnosis (TRIPOD) guidelines and the Declaration of Helsinki were adhered to throughout the execution of this investigation [27,28].

### 2.2. Participants

Using the TQIP database, all adult patients (18 years or older) with isolated severe TBI due to blunt trauma who were registered between 2013 and 2021 were considered for inclusion. An isolated severe TBI was defined as a head AIS ≥ 3, with an AIS ≤ 1 in all other regions. Patients with a head AIS of 6 were excluded, as these injuries are generally not considered survivable.

### 2.3. Outcome

The primary outcome of the study was in-hospital mortality. Patients who were still alive at the time of discharge from the hospital were considered censored. Because the outcome was assessed by external and independent clinicians, blinding was not implemented in the current study. The researchers and statistician in the current study were not involved in the outcome assessment, which helped to minimize the potential bias resulting from non-blinding.

### 2.4. Predictors

Predictors in the current study were patients’ demographics, admission status, as well as comorbidities, and clinical characteristics.

Demographic features consisted of age, sex, race/ethnic origin (White, Black, Asian, American Indian, Pacific Islander, or other), smoking status, payment method (private insurance, government insurance, uninsured), and type of hospital (University, non-teaching, community). Variables pertaining to admission status included oxygen saturation, respiratory rate, body temperature, hypotension (defined as a systolic blood pressure <90 mmHg), tachycardia (defined as a pulse rate >100), and shock index (calculated as the pulse rate divided by the systolic blood pressure) [29], severity of head injury (AIS, 3, 4, or 5), presence of injury in other regions (face, neck, spine, thorax, abdomen, upper extremity, lower extremity, external), level of consciousness (GCS 3–15), presence of intracranial injury (epidural hematoma, traumatic subdural hematoma, traumatic subarachnoid hemorrhage, cerebral contusion, diffuse axonal, or other), neurosurgical intervention (none, within 24 h from admission, or after 24 h from admission), and number of units of packed red blood cells (PRBC) transfused within 4 h from admission (250 mL per unit). Comorbidities and clinical characteristics consisted of previous myocardial infarction, congestive heart failure, coagulopathy, dementia, cerebrovascular disease, diabetes mellitus, chronic renal failure, disseminated cancer, currently receiving chemotherapy for cancer, peripheral vascular disease, chronic obstructive pulmonary disease, alcohol use disorder, drug use disorder, cirrhosis, major psychiatric illness, advanced directives limiting care, and anticoagulant therapy.

### 2.5. Sample Size

The study is a patient-population-register-based study with a total of 545,388 patients. A post hoc power calculation indicated that this sample size has a power of >0.99 to identify a statistically significant area under the receiver operating characteristic curve (AUC) >0.8, at a two-sided *α* level of 0.05.

### 2.6. Missing Data

The multivariate imputation by chained equations algorithm was applied to impute the missing values. Because of the large sample size, only one imputed complete dataset was used for model training and testing.

### 2.7. Statistical Analysis

Patients were grouped based on if they were discharged alive or dead. Continuous variables were presented as means and standard deviations or medians and interquartile ranges, depending on if they were or were not normally distributed. The statistical significance of differences was evaluated using the Student’s *t*-test for the former group and the Mann–Whitney U-test for the latter group. Categorical variables were summarized as counts and percentages, with the Chi-squared test being used to determine the significance of differences. A two-sided *p*-value less than 0.05 was considered statistically significant.

Continuous variables were standardized with a mean of zero and a standard deviation of 1, and multi-nominal variables were converted into multiple dummy variables using the one-hot encoding method before they entered the models.

Penalized Cox regression models with L2 penalty (ridge regression), L1 penalty (LASSO regression), and both penalties (elastic net regression) were used to investigate the associations between the survival outcomes and the predictors in the prediction model. An XGBoost algorithm-powered Cox regression model was trained using the dataset to predict the survival outcome, with a relatively small learning rate (<0.1) to make the boosting process more conservative. The K-fold cross-validation method, with the training dataset split into 5 equal parts, was used throughout the predictor selection and XGBoost-powered model training. The grid search method was used for tuning models’ hyperparameters, including penalty coefficient λ, L1 ratio, and learning rate η, to update the models. The test dataset was used to validate the final model.

Given the imbalance of the survival outcomes (in-hospital mortality only occurred in <10% of patients), a random under-sampling method was used for patients discharged alive to achieve a 1:1 ratio between the patients who died and survived. The above procedure was repeated for 10 under-sampling samples as a sensitivity analysis to validate the robustness of this modelling strategy.

The overall performance of the models was evaluated using the Harrell’s concordance index (C-index), with a value between 0.7 and 0.8 indicating an acceptable model, between 0.8 and 0.9 excellent, and >0.9 outstanding [30]. The time-dependent AUC was used to evaluate the dynamically cumulative performance of the final model [31]. The importance of the predictors in the final model was evaluated using the Shapley additive explanations (SHAP) value [32].

The missing value imputation was conducted in the R statistical programming language, version 4.2.3 (R Foundation for Statistical Computing, Vienna, Austria) using the package *mice* [33]. The penalized Cox regression, XGBoost-powered Cox regression, and model training, test, and evaluation were performed in Python, version 3.9 using the packages *sklearn*, *sksurv*, *lifelines*, *xgboost*, *shap*, and *imblearn* [34].

## 3. Results

### 3.1. Participants

545,388 adult patients were registered in TQIP between 2013 and 2021, after having suffered an isolated severe traumatic brain injury. Patients who died were generally older (74 vs. 67 years old, *p* < 0.001), more often male (64.2% vs. 61.6%, *p* < 0.001), and more likely to be White (78.6% vs. 77.0%, *p* < 0.001) or Asian (3.7 vs. 2.9%, *p* < 0.001). All comorbidities were more common among patients who died in the hospital except for dementia, substance use disorders, and major psychiatric illnesses.

Patients who died were more severely injured (Head AIS 5: 64.4% vs. 15.7%, *p* < 0.001) and consequently tended to have a lower GCS on admission (GCS ≤ 8: 58.9% vs. 7.3%, *p* < 0.001). These patients were also more likely to be hypotensive (6.8% vs. 0.7%, *p* < 0.001) and tachycardic (24.0% vs. 16.2%, *p* < 0.001) on admission. All intracranial injuries were more common among patients who died in the hospital except for epidural hematomas. As a result, patients who died were more likely to have required neurosurgical intervention (19.9% vs. 8.8%, *p* < 0.001) (Table 1).

### 3.2. Model Development

**Figure 1:**
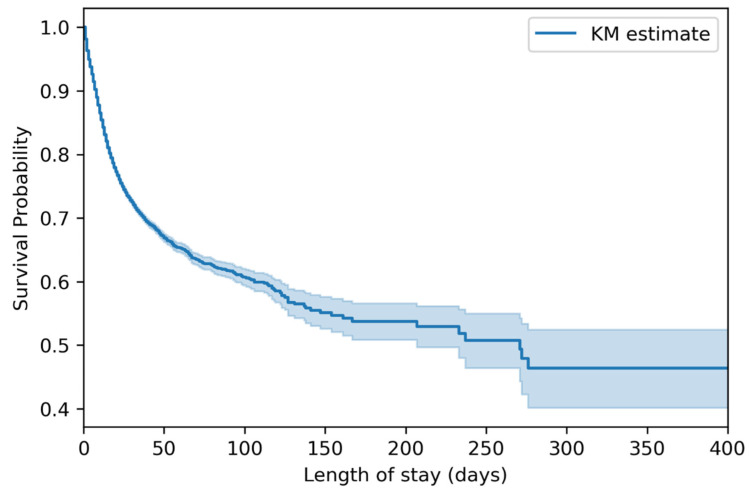
Survival probability of the 545,388 traumatic brain injury patients.

Among the 545,388 patients, the in-hospital mortality rate was 8.6% with a median length of stay of 4 days (Table S1). The survival probability over time is shown in Figure 1.

The Cox regression models with L1 and L2 penalties detected the same top 10 predictors for in-hospital mortality: head AIS 5, hypotension, no neurosurgical intervention, cirrhosis, the presence of an advanced directive limiting care, age, disseminated cancer, epidural hematoma, GCS, and Spine AIS 1 (Supplemental Figures S1–S3).

**Figure 2:**
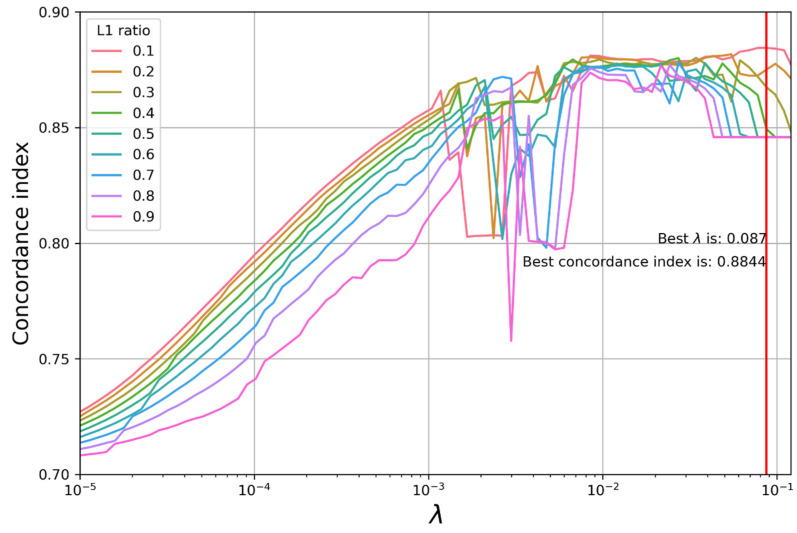
Performance of elastic net Cox regression models with different penalty coefficients (λ) and L1 ratios.

After grid searching, the best performance was found for the elastic net Cox regression model with a penalty coefficient of 0.087, an L1 ratio of 0.1, and a C-index of 0.88 (Figure 2).

**Figure 3:**
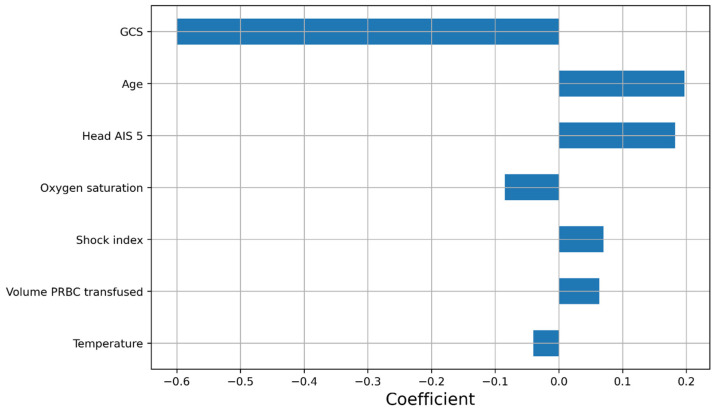
Coefficients of the predictors (standardized value used for continuous variables) in the best elastic net Cox regression model.

In the best elastic net Cox regression model, the predictors with non-zero coefficients were GCS, age, Head AIS 5, oxygen saturation, shock index, volume of PRBC transfused, and temperature (Figure 3).

### 3.3. Predictive Model Specification

Both the top 10 predictors from the L1- or L2-penalized Cox regression models and predictors from the best elastic net Cox regression model were included to train the XGBoost-powered Cox regression model. The model performed excellently in both the training dataset (C-index = 0.8969) and the test dataset (C-index = 0.8963).

**Figure 4:**
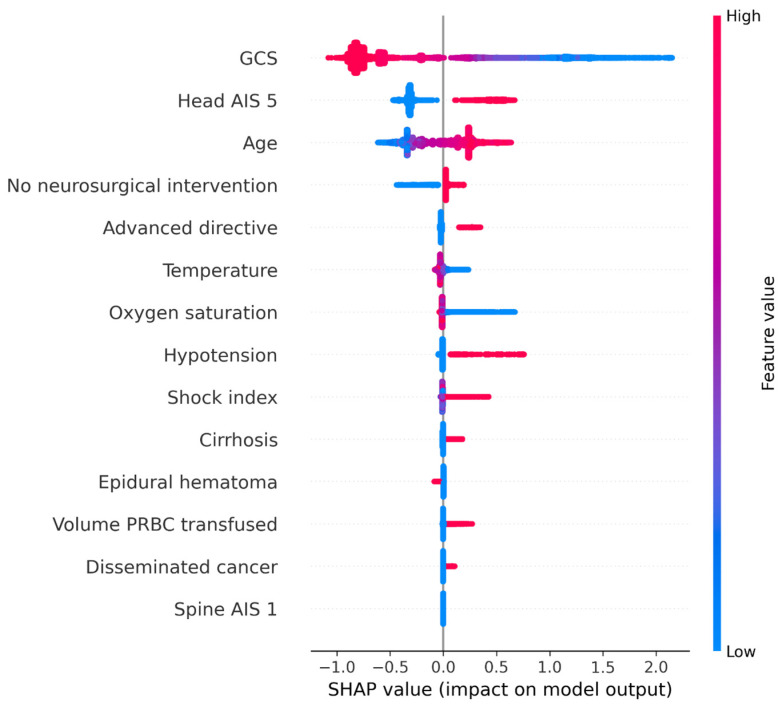
Bee swarm plot of predictors’ impacts on model output. Note: in the Cox regression model, the output is logarithmic value of hazard ratio for the survival outcome.

The effects of the predictors on the model output for the test dataset are shown in Figure 4. A higher GCS was associated with a lower risk of mortality. A Head AIS of 5, older age, no neurosurgical intervention, the presence of an advanced directive limiting care, lower oxygen saturation, hypotension, lower body temperature, higher shock index, cirrhosis, larger blood transfusion, and disseminated cancer were associated with an increased risk of mortality. The importance of an AIS 1 spine injury was negligible.

**Figure 5:**
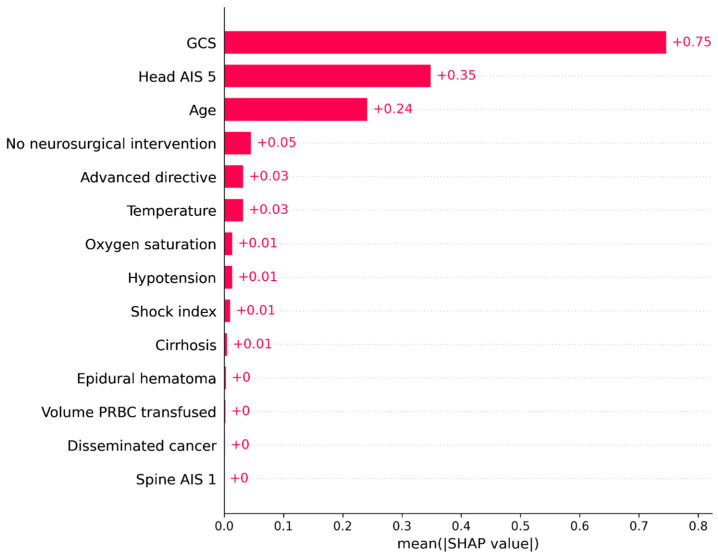
Rank of predictors’ impacts on model output.

The rank of the impact of the predictors according to the mean of the absolute SHAP values is shown in Figure 5. The GCS score is associated with the largest impact on in-hospital mortality, followed by a Head AIS of 5, and age. However, the impacts associated with the other predictors are relatively small and ignorable.

**Figure 6:**
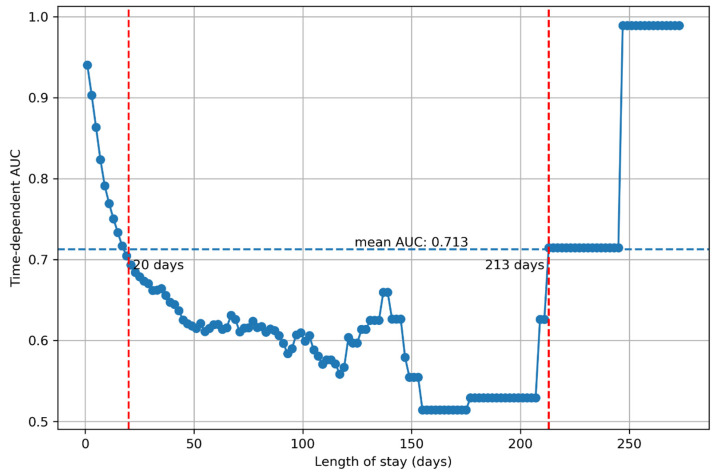
Time-dependent AUC curve of the XGBoost-powered Cox regression model for the test dataset.

The dynamic cumulative performance of the final XGBoost-powered Cox regression model for the test data set is shown in Figure 6. On average, the model performed at an acceptable level for patients with a length of stay of up to 250 days (mean time-dependent AUC = 0.713). However, for patients with a length of stay between 20 and 213 days, the performance of the model was relatively poor (time-dependent AUC < 0.7, Figure 6).

Nevertheless, when limited to patients with a length of stay ≤20 days, which accounts for 95.4% of all the patients, the model achieved an excellent performance (mean time-dependent AUC = 0.813, Supplemental Figure S4). When further limited to patients with a length of stay ≤5 days, which accounts for two-thirds of all the patients, the model achieved an outstanding performance (mean time-dependent AUC = 0.917). The sensitivity analysis resulted in similar results, with a mean C-index of 0.884 (SD = 0.023) and a mean time-dependent AUC of 0.733 (SD = 0.104) for the 10 random under-sampling samples.

## 4. Discussion

XGBoost is a widely recognized machine learning algorithm employed in various supervised learning tasks, encompassing both classification and regression. Integrating XGBoost with the Cox model as decision trees for survival outcomes enables the model to harness the strengths of the gradient boosting algorithm while retaining the interpretability of the Cox model. This hybrid approach has the potential to enhance predictive power compared to traditional Cox regression models.

Time-dependent AUC plays a pivotal role in assessing the predictive performance of a survival model across different time intervals. Unlike the conventional AUC, which evaluates the model’s predictive ability over the entire study period, the time-dependent AUC offers insights into how effectively the model distinguishes individuals who experience an event (e.g., mortality) from those who do not at various time points.

Variable importance measures the relative contribution of each predictor variable (e.g., covariates or features) to the overall predictive capacity of a survival model. It quantifies the influence of each variable on the model’s ability to predict survival outcomes, facilitating the identification of the most influential factors.

In our current study, we employed the XGBoost-powered Cox regression model for patients with severe traumatic brain injury. This model demonstrated outstanding predictive ability for in-hospital mortality during the first 5 days, primarily based on the severity of the injury, the GCS on admission, and the patient’s age. These variables continue to demonstrate an excellent predictive ability up to 20 days after admission, a period of care that accounts for over 95% of severe TBI patients. However, past this cutoff, the model struggles to accurately predict in-hospital mortality using these same variables [30].

Several studies have investigated the top predictors of mortality in patients with severe traumatic brain injury [18,19,20,21,22]. All of these studies agree that age is among the most important predictors [18,19,20,21,22], while all but one also included injury severity [18,19,20,21], as well as GCS [18,19,21,22], in this group. We et al, in particular, also used an XGBoost-powered model and achieved a similar predictive ability with their set of top predictors, which included age, GCS at admission, and the injury severity score for the brain [18]. However, none of these studies considered how the predictive ability of these variables varied over time.

This model provides significant insights into the determinants of mortality after suffering a severe isolated TBI. As is evident from current and previous investigations, non-modifiable risk factors in the form of injury severity, GCS, and age demonstrate the highest predictive ability during the initial period of care, with the model demonstrating an AUC > 0.9 during the first 5 days. This indicates that there appears to be a limit to how much can be done to improve outcomes during this period, beyond those interventions and routines that are already in use. Instead, preventive measures that both reduce the severity and frequency of TBIs are likely of greater importance [35,36,37]. Given the significance of age, targeting these measures toward older and more frail populations who are at the greatest risk of adverse outcomes may be particularly effective [38,39].

However, beyond 20 days, the model struggles to accurately predict outcomes in the ~5% of patients that remain. While the exact cause of this cannot be definitively determined based on the current analysis, this indicates that other factors not captured by the dataset likely become more important for predicting in-hospital mortality after the 20-day cutoff. This could be speculated to be variables related to patient care such as infections and other complications arising in the hospital ward, treatments and medications administered, as well as supportive therapies [40,41,42,43]. This could also indicate that there is a window of opportunity after the initial 20-day period of care, wherein management decisions may significantly influence patient outcomes. There might consequently also be greater opportunities for improving patient outcomes during this period, given the decreasing importance of non-modifiable risk factors in predicting mortality.

Nevertheless, after 210 days the XGBoost-powered Cox regression model improves, with AUCs consistently above 0.7. Given that only a small proportion of the original cohort remains at this stage, significant care should be taken when drawing any conclusions regarding this period. As these AUCs are based on the test dataset, this high predictive ability cannot be attributable to overfitting. Instead, this may suggest an end to the window of opportunity for patients who still require hospital care. Rather than outcomes being determined by patient care, the non-modifiable characteristics of the original injury (AIS, GCS, and age) once again appear to become the primary predictors of mortality. If this is the case, this may function as a suitable threshold for a renewed discussion with patients and their next-of-kin regarding goals of care and continued life-supporting measures.

Predictive modeling has emerged as a valuable tool in healthcare, providing clinicians with a means to predict patient outcomes and allocate resources effectively [11,12]. Neurosurgery, being a complex and high-stakes field, may greatly benefit from the integration of predictive models, particularly those focused on in-hospital mortality [11,12,23,24]. By identifying patients at a higher risk of adverse events, such as mortality, healthcare providers can intervene early, allowing for intensified monitoring, tailored interventions, and closer follow-up, Furthermore, stratifying patients into risk categories enables more efficient resource allocation and optimizes the utilization of healthcare resources. This ensures that patients with a higher predicted risk receive timely care, while low-risk patients can undergo more conservative management approaches, potentially reducing healthcare costs and unnecessary interventions. By considering a patient’s estimated risk of in-hospital mortality, clinicians are better able to weigh the potential benefits and risks of different interventions. At the same time, having this estimate can also enhance shared decision making and empower both patients and their relatives to make informed choices about their care.

This investigation made use of a large, multi-institutional, administrative dataset with over 500,000 patients in order to build a model to predict in-hospital mortality. Furthermore, the predictive ability of over 50 variables could be compared while developing the model. As a result, this study benefits from a relatively high external validity owing to the nature of the sample population. Nevertheless, some limitations need to be addressed. Given the retrospective nature of the dataset, analyses were limited to the variables that had already been recorded. As a consequence, variables such as intracranial pressure, brain arterial pressure, cerebral perfusion pressure, and other vitals measured during the period of care were not available. A more detailed description of preoperative optimization and patient management decisions taken during the hospitalization was also not present in the dataset. It was also not possible to investigate other potential outcomes of interest, such as cause of death, functional outcomes, quality of life, and survival after discharge. Furthermore, while the timing of neurosurgical intervention could be divided into none, within 24 h, and after 24 h, the TQIP dataset lacked the granularity for a more detailed description of the timing. The criteria for intervention were also not captured by TQIP. Finally, it is important to note that the relationships identified are associative rather than causal in nature given the observational study design.

## 5. Conclusions

The XGBoost-powered Cox regression model can achieve an outstanding predictive ability for in-hospital mortality during the first 5 days and continues to demonstrate an excellent predictive ability up to 20 days after admission. During this period of care, which accounts for over 95% of severe TBI patients, in-hospital mortality is chiefly predicted by the severity of the injury, the Glasgow Coma Scale on admission, and the patient’s age. Past 20 days of care, other factors appear to be the primary drivers of in-hospital mortality, indicating a potential window of opportunity for reducing adverse outcomes.

## Figures and Tables

**Table 1:** Characteristics of patients with an isolated severe TBI.

	Survived (N = 498,599)	Died (N = 46,789)	*p*-Value
Length of hospital stay, median [IQR]	4.0 [2.0–7.0]	3.0 [2.0–8.0]	<0.001
Missing, n (%)	3919 (0.8)	2302 (4.9)	
Age, median [IQR]	67 [49–79]	74 [60–82]	<0.001
Sex, n (%)			<0.001
Female	190,692 (38.2)	16,661 (35.6)	
Male	307,239 (61.6)	30,061 (64.2)	
Missing	668 (0.1)	67 (0.1)	
Race, n (%)			<0.001
White	383,863 (77.0)	36,761 (78.6)	
Black	47,635 (9.6)	3514 (7.5)	
Asian	14,670 (2.9)	1750 (3.7)	
American Indian	4693 (0.9)	356 (0.8)	
Pacific islander	1403 (0.3)	151 (0.3)	
Other	35,796 (7.2)	2941 (6.3)	
Missing	6480 (1.3)	644 (1.4)	
History of myocardial infarction, n (%)	8110 (1.6)	1122 (2.4)	<0.001
Congestive heart failure, n (%)	28,071 (5.6)	4749 (10.1)	<0.001
Cerebrovascular disease, n (%)	27,543 (5.5)	3365 (7.2)	<0.001
History of peripheral vascular disease, n (%)	5540 (1.1)	833 (1.8)	<0.001
Diabetes mellitus, n (%)	100,978 (20.3)	11,426 (24.4)	<0.001
Chronic renal failure, n (%)	11,768 (2.4)	2279 (4.9)	<0.001
Dementia, n (%)	44,075 (8.8)	4215 (9.0)	0.222
Coagulopathy, n (%)	32,520 (6.5)	5801 (12.4)	<0.001
Anticoagulant therapy, n (%)	2870 (0.6)	90 (0.2)	<0.001
Missing	100,926 (20.2)	9556 (20.4)	
Currently receiving chemotherapy for cancer, n (%)	3924 (0.8)	800 (1.7)	<0.001
Disseminated cancer, n (%)	5765 (1.2)	1320 (2.8)	<0.001
Current smoker, n (%)	79,330 (15.9)	4177 (8.9)	<0.001
COPD, n (%)	37,049 (7.4)	4496 (9.6)	<0.001
Cirrhosis, n (%)	7658 (1.5)	1790 (3.8)	<0.001
Alcohol use disorder, n (%)	54,543 (10.9)	4868 (10.4)	<0.001
Drug use disorder, n (%)	24,727 (5.0)	1471 (3.1)	<0.001
Major psychiatric illness, n (%)	55,874 (11.2)	3885 (8.3)	<0.001
Advanced directive limiting care, n (%)	22,674 (4.5)	6037 (12.9)	<0.001
Head AIS, n (%)			<0.001
3	283,581 (56.9)	9253 (19.8)	
4	136,610 (27.4)	7405 (15.8)	
5	78,408 (15.7)	30,131 (64.4)	
Face AIS, n (%)			
Injury present	139,844 (28.0)	10,921 (23.3)	<0.001
Neck AIS, n (%)			
Injury present	3099 (0.6)	351 (0.8)	<0.001
Spine AIS, n (%)			
Injury present	5953 (1.2)	158 (0.3)	<0.001
Thorax AIS, n (%)			
Injury present	18,423 (3.7)	2577 (5.5)	<0.001
Abdomen AIS, n (%)			
Injury present	11,363 (2.3)	1574 (3.4)	<0.001
Upper extremity AIS, n (%)			
Injury present	65,262 (13.1)	6416 (13.7)	<0.001
Lower extremity AIS, n (%)			
Injury present	50,267 (10.1)	5309 (11.3)	<0.001
External/Other AIS, n (%)			
Injury present	20,205 (4.1)	2387 (5.1)	<0.001
GCS at admission, n (%)			<0.001
Mild (GCS 14–15)	392,770 (78.8)	11,104 (23.7)	
Moderate (GCS 9–13)	43,050 (8.6)	5986 (12.8)	
Severe (GCS 3–8)	36,331 (7.3)	27,567 (58.9)	
Missing	26,448 (5.3)	2132 (4.6)	
Hypotension at admission, n (%)	3498 (0.7)	3169 (6.8)	<0.001
Missing	12,064 (2.4)	1438 (3.1)	
Tachycardia, n (%)	80,891 (16.2)	11,252 (24.0)	<0.001
Missing	11,639 (2.3)	1195 (2.6)	
Shock index, median [IQR]	0.57 [0.48–0.69]	0.57 [0.45–0.72]	<0.001
Missing, n (%)	13,967 (2.8)	3133 (6.7)	
Oxygen saturation, median [IQR]	98 [96–99]	98 [96–100]	<0.001
Missing, n (%)	25,842 (5.2)	3374 (7.2)	
Respiratory rate, mean (SD)	18.0 (±4.1)	17.3 (±6.9)	<0.001
Missing, n (%)	16,193 (3.2)	3039 (6.5)	
Temperature, mean (SD)	36.6 (±0.9)	36.2 (±1.9)	<0.001
Missing, n (%)	46,866 (9.4)	10,022 (21.4)	
Intracranial injury, n (%)			
Cerebral contusion	125,674 (25.2)	17,584 (37.6)	<0.001
Epidural hematoma	22,534 (4.5)	1855 (4.0)	<0.001
Traumatic subdural hematoma	353,638 (70.9)	36,099 (77.2)	<0.001
Traumatic subarachnoid hemorrhage	155,472 (31.2)	20,948 (44.8)	<0.001
Diffuse axonal injury	5044 (1.0)	1545 (3.3)	<0.001
Other intracranial injury	14,434 (2.9)	3408 (7.3)	<0.001
Neurosurgical intervention, n (%)			<0.001
None	454,615 (91.2)	37,421 (80.0)	
Within 24 h	32,392 (6.5)	8075 (17.3)	
After 24 h	11,324 (2.3)	1218 (2.6)	
Missing	268 (0.1)	75 (0.2)	
Volume PRBC transfused within 4 h, median [IQR]	0.00 [0.00–0.00]	0.00 [0.00–0.00]	<0.001
Missing, n (%)	0 (0.0)	2 (0.0)	
Hospital teaching status, n (%)			<0.001
Community	196,758 (39.5)	17,856 (38.2)	
Non-teaching	87,816 (17.6)	7201 (15.4)	
University	212,138 (42.5)	21,533 (46.0)	
Missing	1887 (0.4)	199 (0.4)	
Payment method, n (%)			<0.001
Private/commercial insurance	128,167 (25.7)	8773 (18.8)	
Medicaid	53,869 (10.8)	3594 (7.7)	
Medicare	237,888 (47.7)	27,564 (58.9)	
Other government insurance	11,854 (2.4)	843 (1.8)	
Self-pay	39,499 (7.9)	3588 (7.7)	
Not billed (for any reason)	1736 (0.3)	125 (0.3)	
Other	11,219 (2.3)	873 (1.9)	
Missing	14,367 (2.9)	1429 (3.1)	

Length of stay is measured in days. Hypotension is defined as a systolic blood pressure < 90 mmHg. Tachycardia is defined as a pulse rate >100. Temperature is measured in degrees Celsius. PRBC transfusion volume is measured in units (250 mL). TBI, traumatic brain injury; COPD, chronic obstructive pulmonary disease; AIS, Abbreviated Injury Scale; GCS, Glasgow Coma Scale.

## Data Availability

The data presented in this study are available upon request.

## Supplementary Materials

The following supporting information can be downloaded at https://www.mdpi.com/article/10.3390/jpm13091401/s1, Table S1: Comparison of the features between the training and test datasets; Figure S1: Coefficients of the predictors from the Ridge Cox regression analysis with varying L2 penalty (top 10 predictors were labeled); Figure S2: Coefficients of the predictors from the LASSO Cox regression analysis with varying L1 penalty (top 10 predictors were labeled); Figure S3: Coefficients of the predictors from the elastic net Cox regression analysis with varying both L1 and L2 penalties (top 10 predictors were labeled); Figure S4: Time-dependent AUC curve of the XGBoost-powered Cox regression model for patients with a length of stay ≤20 days in the test dataset.
