# Supplementary material for: Development and Validation of an XGBoost-Algorithm-Powered Survival Model for Predicting In-Hospital Mortality Based on 545,388 Isolated Severe Traumatic Brain Injury Patients from the TQIP Database
Source: J Pers Med. 2023 Sep 19;13(9):1401. doi: 10.3390/jpm13091401 (PMC10533165; doi:10.3390/jpm13091401)
Supplement: Supplementary file 1 [file jpm-13-01401-s001.zip › jpm-2587860-supplementary.pdf]

Supplemental Materials

Table S1. Comparison of the features between the training and test datasets.

V1	level	Overall (n = 545388)	Training dataset (n = 436421)	Test dataset (n = 108967)	*p*-value
In-hospital mortality (%)		46789 (8.6)	37402 (8.6)	9387 (8.6)	0.644
Length of hospital stay (median [IQR])		4.00 [2.00, 7.00]	4.00 [2.00, 7.00]	4.00 [2.00, 7.00]	0.258
Age (median [IQR])		67.00 [50.00, 79.00]	67.00 [50.00, 79.00]	68.00 [50.00, 79.00]	0.350
Sex (%)	Female	207353 (38.0)	165781 (38.0)	41572 (38.2)	0.216
	Male	337300 (61.8)	270036 (61.9)	67264 (61.7)	
Head AIS (%)	3	292834 (53.7)	233937 (53.6)	58897 (54.1)	0.030
	4	144015 (26.4)	115454 (26.5)	28561 (26.2)	
	5	108539 (19.9)	87030 (19.9)	21509 (19.7)	
GCS (median [IQR])		15.00 [14.00, 15.00]	15.00 [14.00, 15.00]	15.00 [14.00, 15.00]	0.679
Epidural hematoma (%)		24389 (4.5)	19564 (4.5)	4825 (4.4)	0.438
Traumatic subdural hematoma (%)		389737 (71.5)	311858 (71.5)	77879 (71.5)	0.939
Traumatic subarachnoid hemorrhage (%)		176420 (32.3)	141233 (32.4)	35187 (32.3)	0.660
Cerebral contusion (%)		143258 (26.3)	114464 (26.2)	28794 (26.4)	0.188
Diffuse axonal injury (%)		6589 (1.2)	5347 (1.2)	1242 (1.1)	0.022
Other intracranial injury (%)		17842 (3.3)	14303 (3.3)	3539 (3.2)	0.630
Neurosurgical intervention (%)	Missing	343 (0.1)	276 (0.1)	67 (0.1)	0.306
	After 24 hours	12542 (2.3)	10019 (2.3)	2523 (2.3)	
	Within 24 hours	40467 (7.4)	32526 (7.5)	7941 (7.3)	
	None	492036 (90.2)	393600 (90.2)	98436 (90.3)	
Volume PRBC transfused within 4 hours (median [IQR])		0.00 [0.00, 0.00]	0.00 [0.00, 0.00]	0.00 [0.00, 0.00]	0.944
Race (%)					0.083
	White	420624 (77.1)	336451 (77.1)	84173 (77.2)	
	Black	51149 (9.4)	41074 (9.4)	10075 (9.3)	
	Asian	16420 (3.0)	13099 (3.0)	3321 (3.0)	
	American Indian	5049 (0.9)	4115 (0.9)	934 (0.9)	
	Pacific islander	1554 (0.3)	1253 (0.3)	301 (0.3)	
	Other	38737 (7.1)	30944 (7.1)	7793 (7.2)	
	Missing	7124 (1.3)	5704 (1.3)	1420 (1.3)	

Anticoagulant_therapy (%)		2960 (0.5)	2374 (0.5)	586 (0.5)	0.690
History of myocardial infarction (%)		9232 (1.7)	7328 (1.7)	1904 (1.7)	0.122
Congestive hhear failure (%)		32820 (6.0)	26254 (6.0)	6566 (6.0)	0.908
Coagulopathy (%)		38321 (7.0)	30639 (7.0)	7682 (7.0)	0.740
Alcohol use disorder (%)		59411 (10.9)	47533 (10.9)	11878 (10.9)	0.936
Dementia (%)		48290 (8.9)	38635 (8.9)	9655 (8.9)	0.940
Disseminated cancer (%)		7085 (1.3)	5628 (1.3)	1457 (1.3)	0.221
Cerebrovascular disease (%)		30908 (5.7)	24710 (5.7)	6198 (5.7)	0.745
Diabetes mellitus (%)		112404 (20.6)	89928 (20.6)	22476 (20.6)	0.884
Chronic renal failure (%)		14047 (2.6)	11251 (2.6)	2796 (2.6)	0.830
Currently receiving chemotherapy for cancer (%)		4724 (0.9)	3767 (0.9)	957 (0.9)	0.644
History of peripheral vascular disease (%)		6373 (1.2)	5085 (1.2)	1288 (1.2)	0.655
Chronic obstructive pulmonary disease (%)		41545 (7.6)	33154 (7.6)	8391 (7.7)	0.251
Drug use disorder (%)		26198 (4.8)	20971 (4.8)	5227 (4.8)	0.914
Major psychiatric illness (%)		59759 (11.0)	47889 (11.0)	11870 (10.9)	0.453
Advanced directive limiting care (%)		28711 (5.3)	22983 (5.3)	5728 (5.3)	0.905
Cirrhosis (%)		9448 (1.7)	7583 (1.7)	1865 (1.7)	0.565
Current smoker (%)		83507 (15.3)	66668 (15.3)	16839 (15.5)	0.147
Hypotension (%)		6667 (1.2)	5317 (1.2)	1350 (1.2)	0.297
Ttachycardia (%)		92143 (16.9)	73595 (16.9)	18548 (17.0)	0.454
Shock index (median [IQR])		0.57 [0.47, 0.69]	0.57 [0.47, 0.69]	0.57 [0.47, 0.69]	0.472
Hospital teaching status (%)	Missing	2086 (0.4)	1660 (0.4)	426 (0.4)	0.549
	Community	214614 (39.4)	171639 (39.3)	42975 (39.4)	
	Non-Teaching	95017 (17.4)	75941 (17.4)	19076 (17.5)	
	University	233671 (42.8)	187181 (42.9)	46490 (42.7)	
Payment method (%)	Missing	15796 (2.9)	12631 (2.9)	3165 (2.9)	0.997
	Medicare or Medicaid	322915 (59.2)	258377 (59.2)	64538 (59.2)	
	Not Billed (for any reason)	1861 (0.3)	1481 (0.3)	380 (0.3)	
	Other	12092 (2.2)	9674 (2.2)	2418 (2.2)	
	Other Government Insurance	12697 (2.3)	10141 (2.3)	2556 (2.3)	
	Private/Commercial Insurance	136940 (25.1)	109633 (25.1)	27307 (25.1)	
	Self-Pay	43087 (7.9)	34484 (7.9)	8603 (7.9)	
Oxygen saturation (median [IQR])		98.00 [96.00, 99.00]	98.00 [96.00, 99.00]	98.00 [96.00, 99.00]	0.845
Respiratory rate (median [IQR])		18.00 [16.00, 20.00]	18.00 [16.00, 20.00]	18.00 [16.00, 20.00]	0.049

Temperature (median [IQR])		36.70 [36.40, 36.90]	36.70 [36.40, 36.90]	36.70 [36.40, 36.90]	0.571
Injury present: face (%)		150765 (27.6)	120594 (27.6)	30171 (27.7)	0.716
Injury present: neck (%)		3450 (0.6)	2739 (0.6)	711 (0.7)	0.365
Injury present: spine (%)		6111 (1.1)	4802 (1.1)	1309 (1.2)	0.005
Injury present: thorax (%)		21000 (3.9)	16732 (3.8)	4268 (3.9)	0.207
Injury present: abdomen (%)		12937 (2.4)	10302 (2.4)	2635 (2.4)	0.268
Injury present: upper extremity (%)		71678 (13.1)	57382 (13.1)	14296 (13.1)	0.806
Injury present: lower_extremity (%)		55576 (10.2)	44397 (10.2)	11179 (10.3)	0.404
Injury present: external (%)	1	22592 (4.1)	18065 (.1)	4527 (4.2)	0.829

Student t-tests were used for normally distributed variables, Mann-Whitney U tests for non-normally distributed variables, and chi-squared tests for categorical variables.

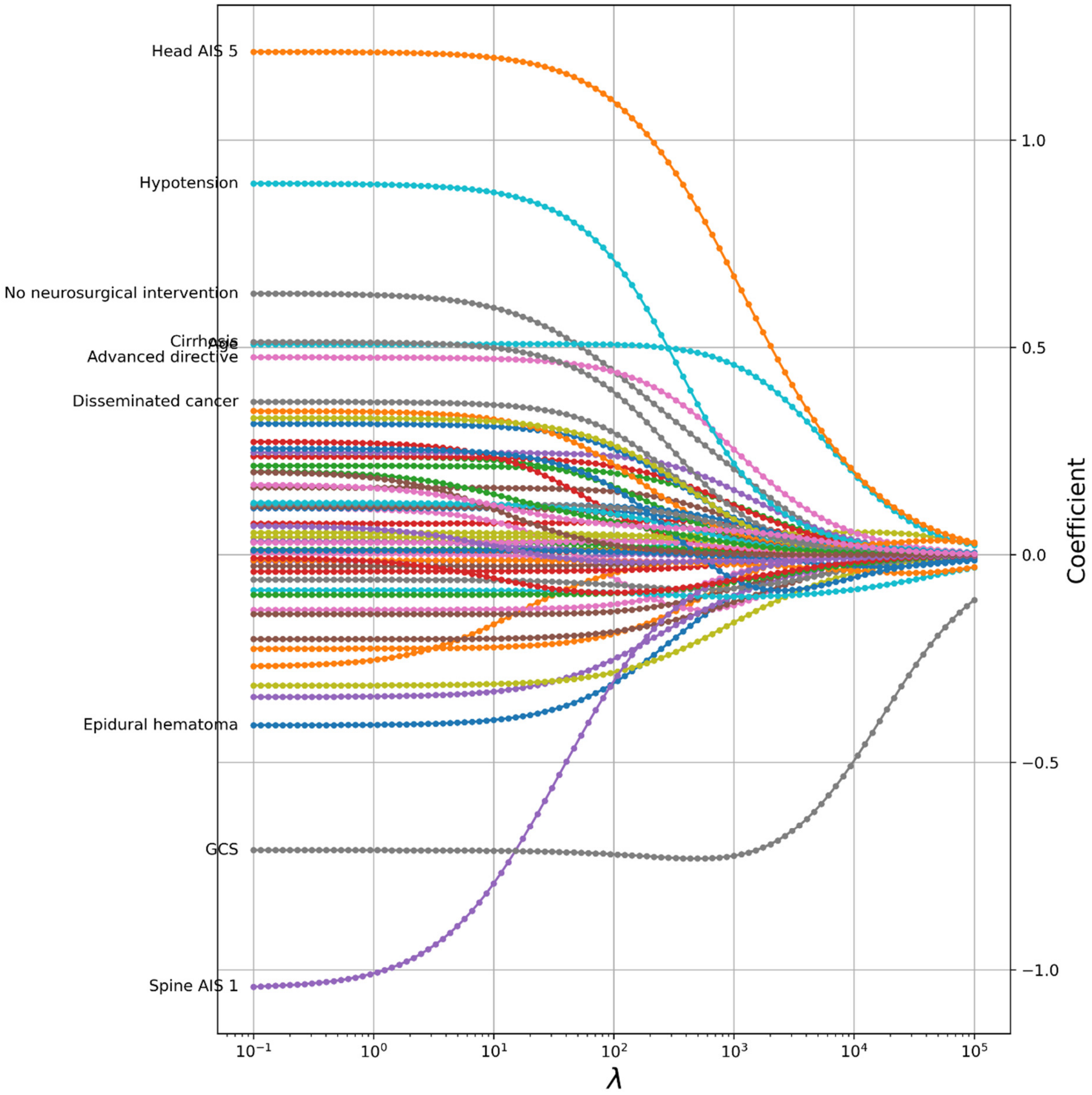

Figure S1. Coefficients of the predictors from the Ridge Cox regression analysis with varying L2 penalty (top 10 predictors were labeled).

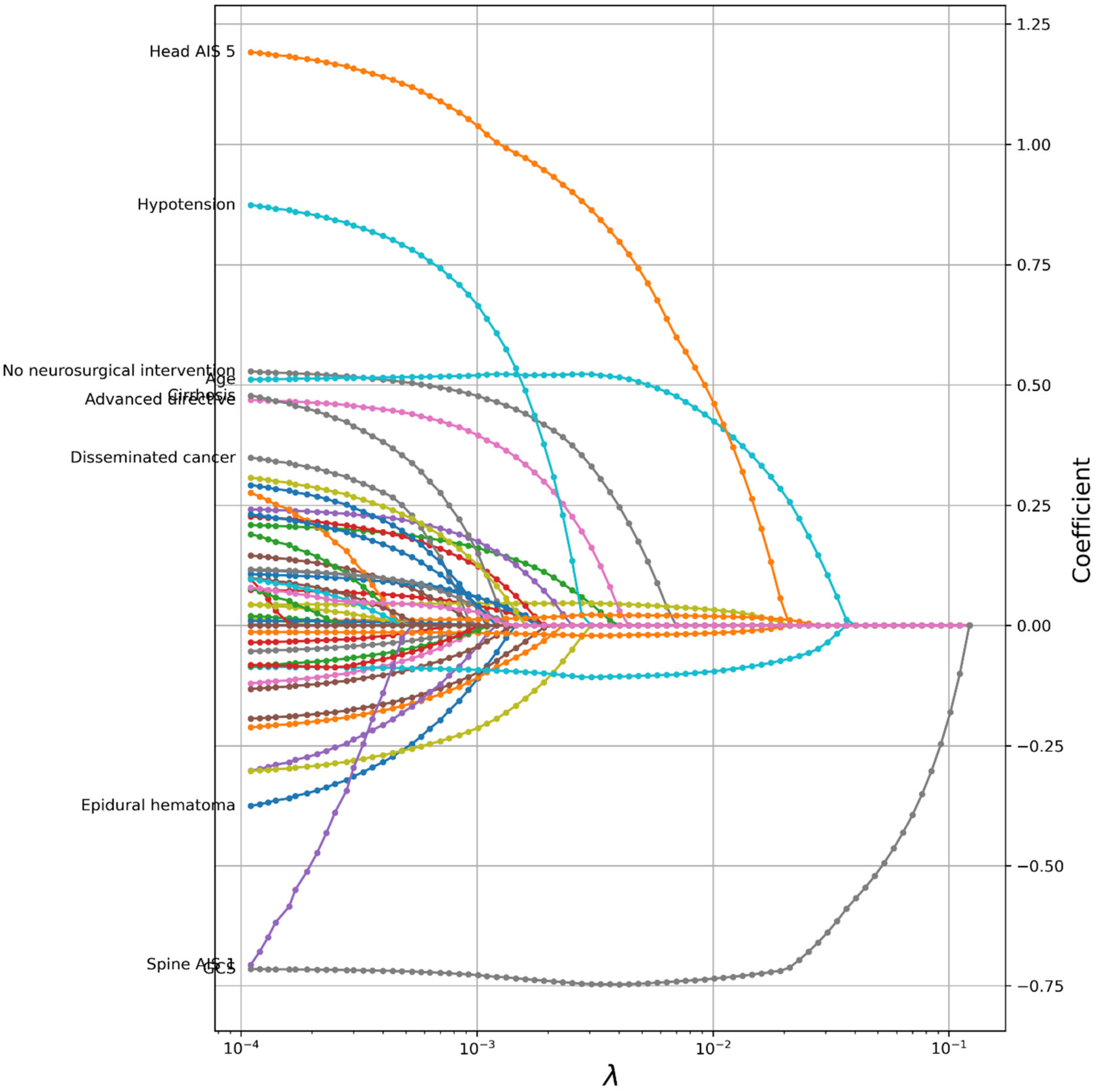

Figure S2. Coefficients of the predictors from the LASSO Cox regression analysis with varying L1 penalty (top 10 predictors were labeled).

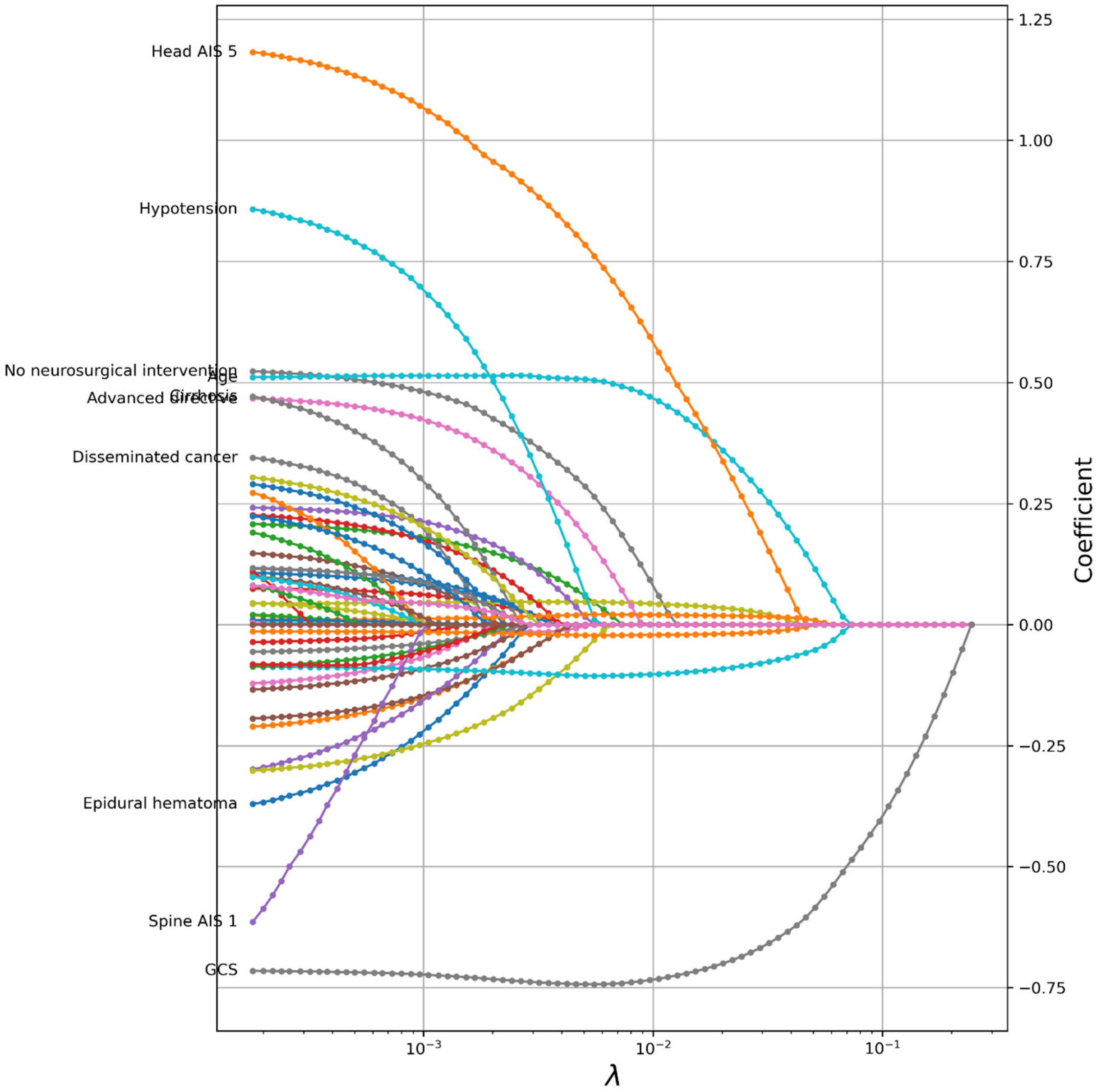

Figure S3. Coefficients of the predictors from the elastic net Cox regression analysis with varying both L1 and L2 penalties (top 10 predictors were labeled).

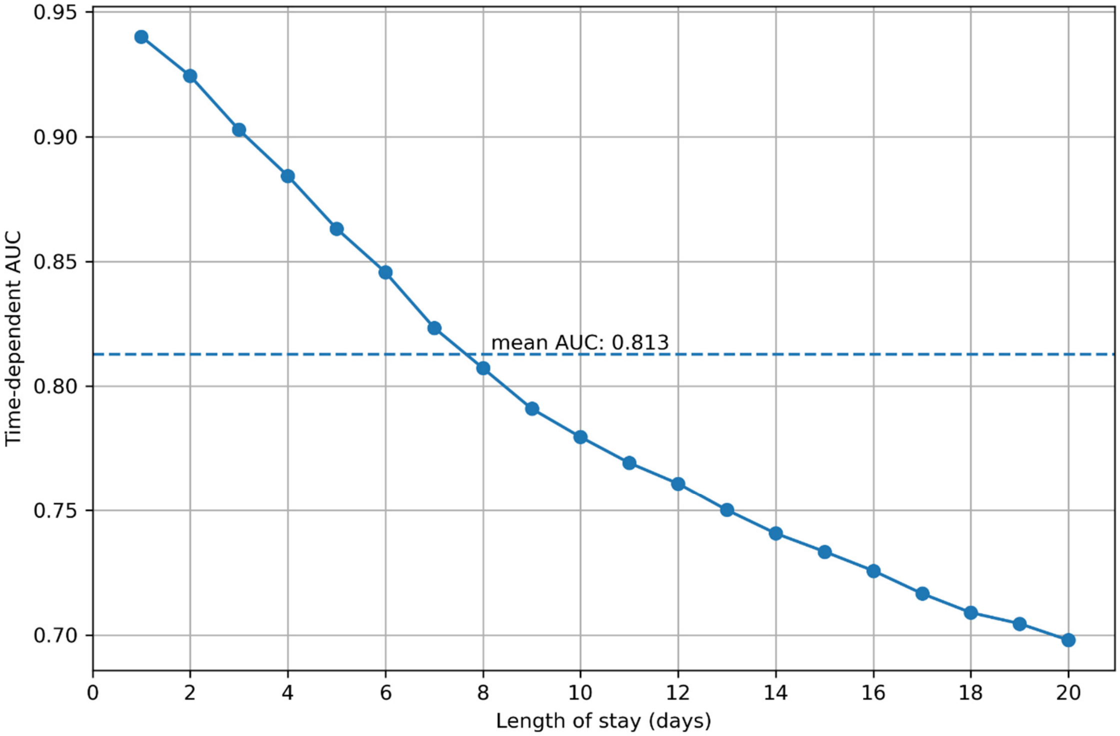

Figure S4. Time-dependent AUC curve of the XGBoost-powered Cox regression model for patients with a length of stay <=20 days in the test dataset.
